# A novel targeted/untargeted GC-Orbitrap metabolomics methodology applied to *Candida albicans* and *Staphylococcus aureus* biofilms

**DOI:** 10.1007/s11306-016-1134-2

**Published:** 2016-11-05

**Authors:** Stefan Weidt, Jennifer Haggarty, Ryan Kean, Cristian I. Cojocariu, Paul J. Silcock, Ranjith Rajendran, Gordon Ramage, Karl E. V. Burgess

**Affiliations:** 1 Polyomics, University of Glasgow, 211 Wolfson Wohl Translational Cancer Research Centre, Garscube Campus, Glasgow, G61 1QH UK; 2 Oral Sciences Research Group, Glasgow Dental School, College of Medical, Veterinary and Life Sciences, University of Glasgow, Glasgow, UK; 3 ThermoFisher Scientific, Hemel Hempstead, UK

**Keywords:** Metabolomics, *Candida*, *Staphylococcus*, Biofilm

## Abstract

**Introduction:**

Combined infections from *Candida albicans* and *Staphylococcus aureus* are a leading cause of death in the developed world. Evidence suggests that *Candida* enhances the virulence of *Staphylococcus*—hyphae penetrate through tissue barriers, while *S. aureus* tightly associates with the hyphae to obtain entry to the host organism. Indeed, in a biofilm state, *C. albicans* enhances the antimicrobial resistance characteristics of *S. aureus*. The association of these microorganisms is also associated with significantly increased morbidity and mortality. Due to this tight association we hypothesised that metabolic effects were also in evidence.

**Objectives:**

To explore the interaction, we used a novel GC-Orbitrap-based mass spectrometer, the Q Exactive GC, which combines the high peak capacity and chromatographic resolution of gas chromatography with the sub-ppm mass accuracy of an Orbitrap system. This allows the capability to leverage the widely available electron ionisation libraries for untargeted applications, along with expanding accurate mass libraries and targeted matches based around authentic standards.

**Methods:**

Optimised *C. albicans* and *S. aureus* mono- and co-cultured biofilms were analysed using the new instrument in addition to the fresh and spent bacterial growth media.

**Results:**

The targeted analysis experiment was based around 36 sugars and sugar phosphates, 22 amino acids and five organic acids. Untargeted analysis resulted in the detection of 465 features from fresh and spent medium and 405 from biofilm samples. Three significantly changing compounds that matched to high scoring library fragment patterns were chosen for validation.

**Conclusion:**

Evaluation of the results demonstrates that the Q Exactive GC is suitable for metabolomics analysis using a targeted/untargeted methodology. Many of the results were as expected: e.g. rapid consumption of glucose and fructose from the medium regardless of the cell type. Modulation of sugar-phosphate levels also suggest that the pentose phosphate pathway could be enhanced in the cells from co-cultured biofilms. Untargeted metabolomics results suggested significant production of cell-wall biosynthesis components and the consumption of non-proteinaceous amino-acids.

**Electronic supplementary material:**

The online version of this article (doi:10.1007/s11306-016-1134-2) contains supplementary material, which is available to authorized users.

## Introduction

Metabolomics is now an established methodology for the analysis of small biomolecules. Applications for metabolomics include both hypothesis generating and hypothesis testing experiments, the former to generate new research questions, and the latter to provide a focused analysis on a subset of metabolites with greater precision and sensitivity. Mass spectrometry is now the most commonly used detection methodology for metabolomics, due to its superior selectivity, sensitivity and range of analytes detected. Modern instruments, such as Orbitrap-based mass spectrometers, are capable of sub-ppm mass accuracy at chromatographic timescales, allowing calculation of predicted molecular formula based on the mass defect of a detected ion (Kind and Fiehn 2007), and ultimately facilitating metabolite annotation. To enhance the capabilities of the instrument, an MS is normally connected to a separation system to reduce the complexity of biological samples prior to their detection. The two most common separation methods applied in metabolomics are gas and liquid chromatography, although some groups, notably the Soga group (Soga et al. 2003) have successfully applied CE-MS.

Gas chromatography (GC) provides high chromatographic resolution and increased peak capacity for small molecules as compared to liquid chromatography (LC) (Lei et al. 2011), but has hitherto not been coupled to Orbitrap mass spectrometry in a commercial instrument. Hyphenated methods that combine GC with analytical techniques such as mass spectrometry have been previously used for metabolite detection and characterization. GC–MS is frequently applied to metabolomics, including the analysis of pathogens e.g. (MacRae et al. 2013; Ansong et al. 2013; Adam et al. 2002; Kamthan et al. 2012).

Orbitrap based high resolution accurate mass instrumentation offers the ability to obtain mass spectral data at high resolving power with mass accuracies <1 ppm. This is useful in the context of metabolite profiling where the ultimate goal is often confident compound identification and quantification. In this study, a novel GC–MS system, namely: the Thermo Scientific Q Exactive GC coupled to a GC with PTV and split/splitless injector (Thermo Scientific™ TRACE™ 1310 GC) was used. This allows to scan rate of 7.4 Hz at 60,000 resolution (FWHM at *m/z* 200), providing >20 datapoints across a typical GC peak (Peterson et al. 2010). Although untargeted analysis using GC–MS is less common, this approach is generally applied to clinical datasets. The Q Exactive GC provides a unique resource for the untargeted analysis of pathogens, however, one of the limitations of untargeted GC–MS analysis is the requirement for library matches and the propensity for common fragments to be observed. For this reason the application of filtering mechanisms to specify, for example, only trimethylsilane-derivitised compounds is beneficial in reducing false positives. An additional method, used successfully by our group (Creek et al. 2011) in LCMS is a combination of targeted analysis, where retention time standards are used to confirm identifications of core metabolites at the same time as unbiased analysis of the rest of the dataset is performed. We have applied this to the analysis of two pathogenic organisms: *Staphylococcus aureus* and *Candida albicans*.

The interkingdom interaction between *Staphylococcus aureus* and *Candida albicans* is not uncommon as these are frequently been shown to coexist within the human host as complex biofilm communities (Adam et al. 2002; Monroy et al. 2005; O’Donnell et al. 2015). These pathogens, both leading pathogens in bloodstream and systemic infections and a major cause of morbidity and mortality in hospitalized patients, are of significant interest because of the escalating development of antimicrobial resistance and the increasing involvement polymicrobial biofilms in chronic and systemic infections (Perlroth et al. 2007). These prokaryotes and eukaryotes have been shown to co-aggregate together (Peters et al. 2012a, b), existing within a dynamic and interactive state (Shirtliff et al. 2009). It appears that in an analogous manner to *C. albicans* and oral streptococci biofilms (Adam et al. 2002), yeast cells have the capacity to modulate the action of antibacterial agents and staphylococci, which can also affect the activity of antifungal agents in these biofilms. For example, it has been reported that the presence of *C. albicans* along with *S. aureus* protects against vancomycin treatment in concentrations as high as 1600 mg/mL (Harriott and Noverr 2010), which has been suggested to be a multifactorial process (Harriott and Noverr 2009). Importantly, their interaction has been associated with enhanced pathogenic behaviour, disease severity and morbidity (Nair et al. 2014).

Experimentally, there have been some interesting mechanistic observations. For example, it has been shown that *S. aureus* preferentially adhere to hyphal elements of *C. albicans* (Peters et al. 2010), relying on the adhesion to the aglutanin-like sequence 3 protein (Als3p) from *C. albicans* to adhere to its hyphae (Peters et al. 2012b), though it is likely that other proteins are involved. It is thought that adhesion to hyphae, one of *C. albicans* most influential virulence factors, may assist *S. aureus* in penetrating into the host, a manner analogous to injection from a needle-stick injury. This has been demonstrated in mouse studies, where in mixed infections those mice infected with *C. albicans Δals3* strains along *S. aureus* were unable to invade the tongue, whereas the wild type infections demonstrated co-infection (Peters et al. 2012b). The ramifications of this enhanced invasive capacity have been shown historically to impact mortality, where synergism between the co-infection intraperitoneally in a mouse model was shown to lead to 100 % mortality, whereas mono-species infections caused no mortality whatsoever (Carlson 1983). Whether or not the relationship between the two organisms is physical or chemical remains to be determined, though there is evidence that growth related synergy is an important factor in their co-habitation of micro-niches (Carlson and Johnson 1985).

We demonstrate that the Q Exactive-GC instrument allows analysis of eukaryotic and prokaryotic metabolomes at high resolution, allowing us to probe for interkingdom metabolic interactions. We apply the instrument to the analysis of *C. albicans* and *S. aureus* biofilms and combine information from a targeted and untargeted approach.

## Experimental

### Chemicals and reagents

All chemicals and standards were purchased from Sigma-Aldrich unless otherwise stated. N-Methyl-N-(trimethylsilyl) trifluoroacetamide (MSTFA) and trimethylchlorosilane (TMCS) were purchased from Thermo Scientific. LC–MS grade Water (H_2_O) and Methanol (CH_3_OH) were purchased from Rathburn Chemicals Ltd. Chloroform (CHCl_3_) analytical grade was purchased from Fisher Chemicals.

### *Candida* and *Staphylococcus* stains and culture conditions

*Candida albicans* laboratory strain SC5314 (ATCC MYA-2876) was used in this study. *C. albicans* was sub-cultured on Sabouraud’s dextrose (SAB) agar (Fluka Analytical, India) and grown at 37 °C for 48 h and maintained at 4 °C. Broths were prepared by inoculating yeast peptone dextrose (YPD) medium (Sigma Life Sciences, USA), followed by incubation at 37 °C, at 188 rpm, for 16-18 h. *Staphylococcus aureus* Newman strain (NCTC 8178/ATCC) was cultured on Luria–Bertani (LB) agar (Sigma Life Sciences, USA) and incubated for 48 h at 37 °C before being maintained at 4 °C. Overnight cultures were incubated aerobically in LB broth (Sigma Life Sciences, USA) in a shaking incubator set to 37 °C, at a speed of 188 rpm, for 16-18 h.

### Biofilm culture conditions

Overnight cultures of *C. albicans* and *S. aureus* were centrifuged at 3000 rpm for 5 min (Heraeus Megafuge 16R, Thermo Fisher Scientific, Germany) and washed twice using sterile phosphate buffered saline (PBS) (Fisher Chemicals, Leicestershire, UK). *C. albicans* and *S. aureus* were standardised to 10^6^ cells/mL in 50 % foetal bovine serum (FBS). 50 % FBS with no inoculum was used as a control. 1 mL of each of the individual cultures, mixed-cultures and controls were added to individual wells on a 24-well plate (Costar 3524 well plate, Corning Incorporated, NY, USA) containing a sterile cell culture Thermanox cover slip (13 mm Ø, Thermo Fisher Scientific, NY, USA) The cultures were incubated aerobically at 37 °C for 24 h. This experiment utilized six technical replicates for each condition.

### Extracellular-metabolite extraction

Medium controls (50 % FBS with MilliQ water) and biofilm supernatants were transferred into reaction tubes (1.5 mL, Greiner bio-one GmbH, Germany) containing ice cold chloroform:methanol:water (1:3:1 v:v:v) at a ratio of 1:40 (sample:extraction solvent). The reaction tubes were vortexed at maximum speed for 15 s (Vortex Genie 2, Scientific Industries, NY, USA) and stored at -80 °C prior to derivatization and analysis.

### Intracellular-metabolite extraction

Cell culture cover slips were removed from the wells and placed in bijouxs containing ice cold 10 mM ammonium bicarbonate. The bijouxs were sonicated at maximum speed for 10 min at 4 °C (XUB5, 4.5 L, Grant Instruments, Cambridgeshire, UK). The supernatants, including the debris, were removed and placed in clean reaction tubes. The biofilm suspensions were centrifuged at 3000 rpm for 5 min at 4 °C, the supernatants were removed, and 1 mL of a 1 mL:1 g extraction solvent (ChCl_3_:MeOH:H_2_O 1:3:1 v:v:v): 0.1 mm acid washed glass beads (Sigma Aldrich, USA) were added to each pellet. Bead beating was carried out at 3000 rpm for 10 min at 4 °C (Disrupter Genie, Scientific Industries, NY, USA). The samples were centrifuged at 13,000 rpm for 5 min at 4 °C, the supernatants were removed and stored at -80 °C prior to derivatization and analysis.

### Sample preparation and instrument parameters

Sample preparation for GC–MS was performed as described in detail in Supplementary Methods, but in brief, consisted of drying to completeness followed by methoxymation and trimethylsilylation, prior to injection on a Q Exactive GC. A total run time of 24.5 min was applied. Instrument parameters and gradient details were configured for the Q Exactive GC as described in supplementary methods.

### Comparison with ITQ-900

Foetal Calf Serum (PAA, Pasching, Austria) was extracted by addition of methanol (FCS:MeOH 1:9), vortexing (10 s) and centrifugation (13,000 g, 15 min). 150 µL aliquot of FCS extract was dried in vial insert using a Savant SPD1010 SpeedVac concentrator (Thermo Scientific) for 60 min. Samples were then prepared for GC–MS using an ITQ-900 as described in supplementary methods.

### Data processing

Acquired data was processed using Tracefinder 4.0 (Thermo Fisher Scientific, Runcorn, UK) for targeted analysis, and the XCMS/MzMatch/IDEOM software (Smith et al. 2006; Scheltema et al. 2011; Creek et al. 2012) for untargeted analysis. For sensitivity comparison purposes, Tracefinder 4.0 was also used to obtain feature sets from the ITQ900 and Q-Exactive GC. Further details, CAS numbers for detected compounds and MSI classification according to (Sumner et al. 2014) was applied for all targeted compounds and is included in the Supplementary Methods and Data. Features detected from the untargeted analysis are included in Supplementary Data 2 for intracellular compounds and Supplementary Data 3 for extracellular compounds.

### Scanning electron microscopy

*C. albicans* and *S. aureus* polymicrobial biofilm was standardised and grown in 50 % FBS for 24 h on Thermanox™ coverslips (Nunc, Roskilde, Denmark). After incubation, media was removed and coverslips were washed in PBS to remove non-adherent cells. After washing, biofilms were fixed and processed for SEM as previously described (Erlandsen et al. 2004). In brief, biofilms were fixed using 2 % glutaraldehyde, 2 % paraformaldehyde and 0.15 % w/v alcian blue in 0.15 M sodium cacodylate before being sputter coated in gold and imaged at a magnification of ×2000 using a JEOL JSM-6400 scanning electron microscope.

## Results and discussion

**Fig. 1:**
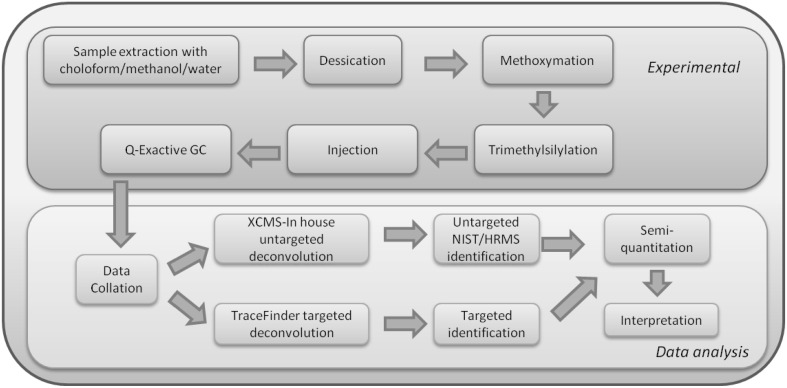
Workflow diagram highlighting the main steps in the hybrid targeted/untargeted workflow. Sample preparation and data acquisition (in *red*) steps followed by data processing and results interpretation (in *blue*). Samples are extracted using a chloroform/methanol/water mixture, as described in *methods*, followed by dessication in a centrifugal evaporator. Derivitisation consists of methoxymation followed by trimethylsilylation and the resulting sample is then injected onto the instrument. Data, once collected, is analysed either using a targeted platform, or an untargeted, XCMS-based platform

The method was optimised for broad detection of bacterial metabolites using the workflow in Fig. 1. To normalise for potential analytical error related to derivatization efficiency and injection volume, standards were added during sample preparation as described in Supplementary Table S1.

### Optimisation of the method

The targeted method was optimised for the detection of 36 sugars and sugar phosphates (see Supplementary Table S2), 22 amino acids (see Supplementary Table S3), and five organic acids (see Supplementary Table S4), for a total of 63 compounds. The most intense fragment was selected for detection of each of the compounds.

### Method performance

Most GC–MS metabolomics methods follow a two-step derivatization technique that involves methoximation followed by silylation, but reagent quantities, incubation temperature and timing vary from lab to lab. In this study, the protocol consisted of 50 µL of 20 mg/ml (w/v) methoxyamine HCl in pyridine incubated at 60 °C for 120 min with silylation performed by incubation at 80 °C for a further 120 min. Overall, short derivatisation methods described in such seminal work as those by Kind and Dunn (Kind et al. 2009; Dunn et al. 2011) suit rapid turnaround for high throughput applications, but more consistent results may be obtained from a long incubation. Recent literature suggests, however, that more consistent results at the sacrifice of metabolite scope may be obtained by substituting alkylation for the more commonly used silylation derivatisation (Villas-Bôas et al. 2011).

### Comparison with pre-existing instrumentation

**Fig. 2:**
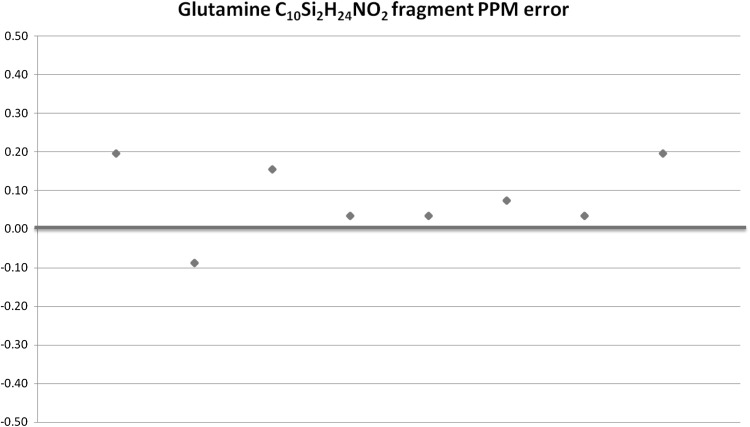
PPM error of the 246.1340 m/z (C_10_Si_2_H_24_NO_2_) reporter ion from Glutamine across the detected peak. Note that all masses are within 300 ppb of the true mass

**Fig. 3:**
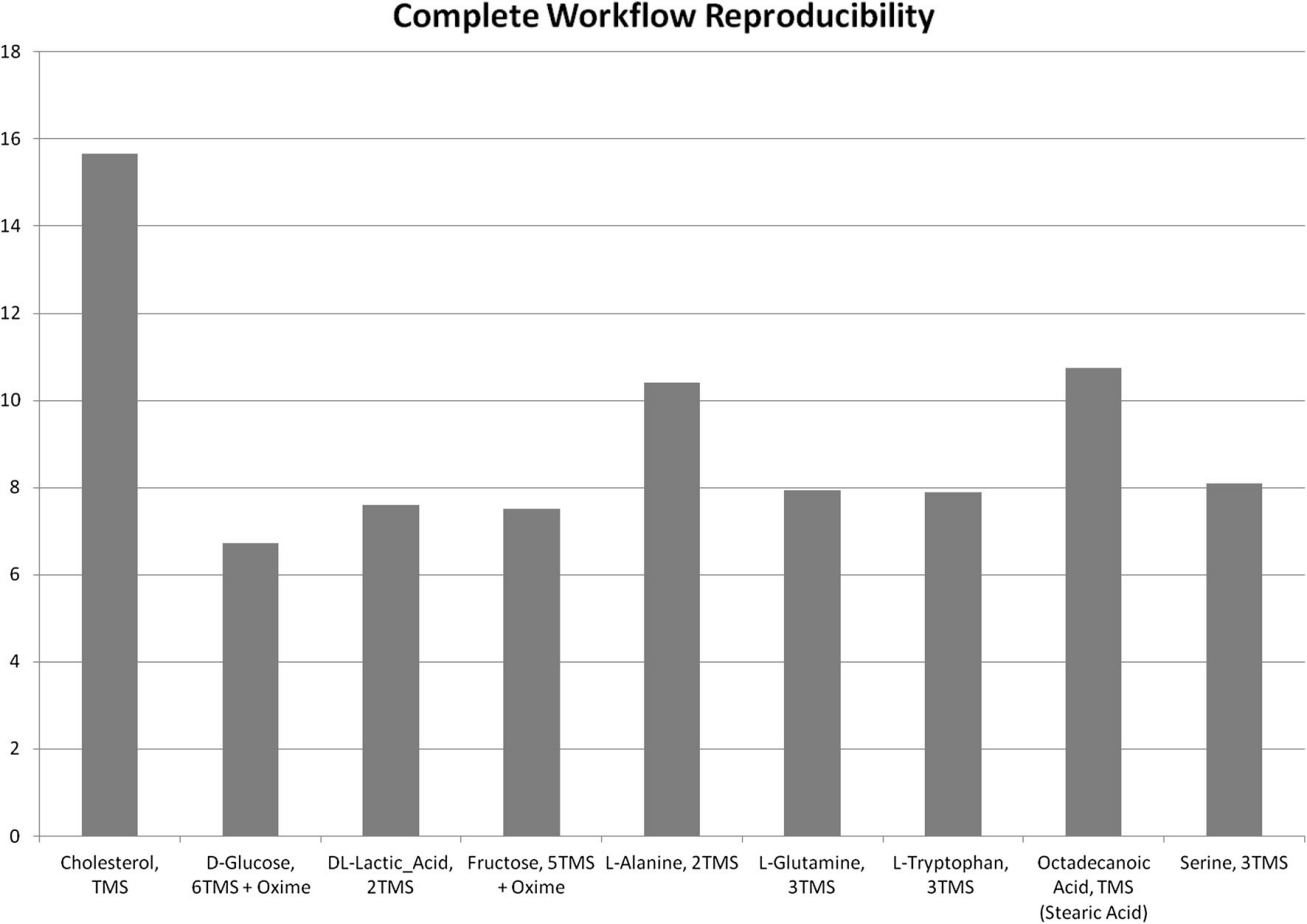
Reproducibility of nine compounds from serum (the growth medium for the bacterial, yeast and co-cultures) providing a measure of the reproducibility of sample analysis and preparation measured by relative standard deviation. Each sample was processed separately to demonstrate that the entire analytical process, from sample extraction, through derivatisation and injection, to analysis, is highly consistent

To perform a general comparison of the capabilities of the GC Q-Exactive against conventional GCMS instrumentation, we ran identical derivitised serum samples (bacterial and yeast growth medium) on both the GC Q-Exactive and an ITQ-900 with TRACE ULTRA GC system. Using the TraceFinder 4.0 software, deconvolution resulted in the detection of 856 features in the case of the GC Q-Exactive, compared to 179 in the case of the ITQ, demonstrating the enhanced selectivity provided by the high resolution of the Orbitrap and the increased sensitivity characteristic of the instrument. We also assessed mass accuracy by plotting the ppm error of the quantitation ion from the amino acid glutamine. The systematic error is around 0.3 ppm and the RSD is 1.75 × 10^−5^ %, demonstrating high mass accuracy across the peak (see Fig. 2). Reproducibility is also excellent, based on injection of the same sample derivatised and injected independently (RSDs of all selected compounds <15 %, except for cholesterol at 16 %, see Fig. 3). This demonstrates conformity to the standards for analysis described in the HUSERMET study as being essential for clinical and pharmaceutical applications (Dunn et al. 2011).

### *Candida albicans* and *Staphylococcus aureus* mono- and co-culture metabolomics

**Fig. 4:**
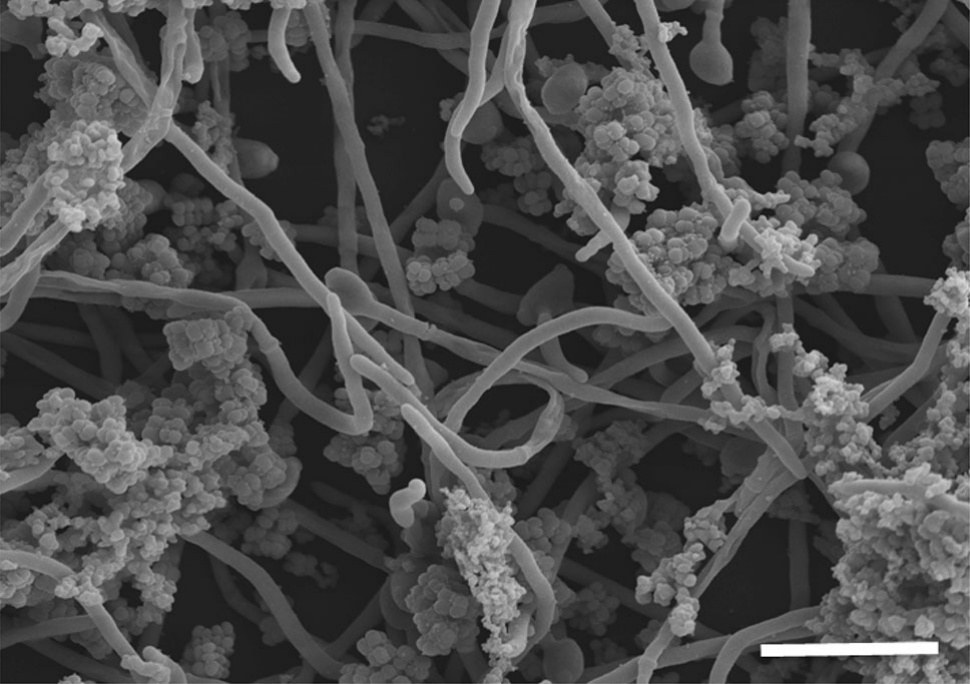
SEM of *Candida albicans* (hyphae) and *Staphylococcus aureus* (spheroids) co-culture. Note the tight association between the *S. aureus* cells and the fungal hyphae. Note also the presence of extracellular matrix coating and secreted from the *S. aureus*. Scale bar corresponds to 100 µm

Scanning electron micrographs showed tight association between *C. albicans* hyphae and *S. aureus* in co-culture conditions (Fig. 4). Targeted analysis with confident identifications based on a panel of authentic standards formed the basis of the analytical method. Additional annotations resulting from the untargeted analysis using the deconvolution features of MzMatch allowed us to expand the coverage of pathways of interest, as well as detect unexpected compounds that could form the basis of new hypotheses to test.

**Fig. 5:**
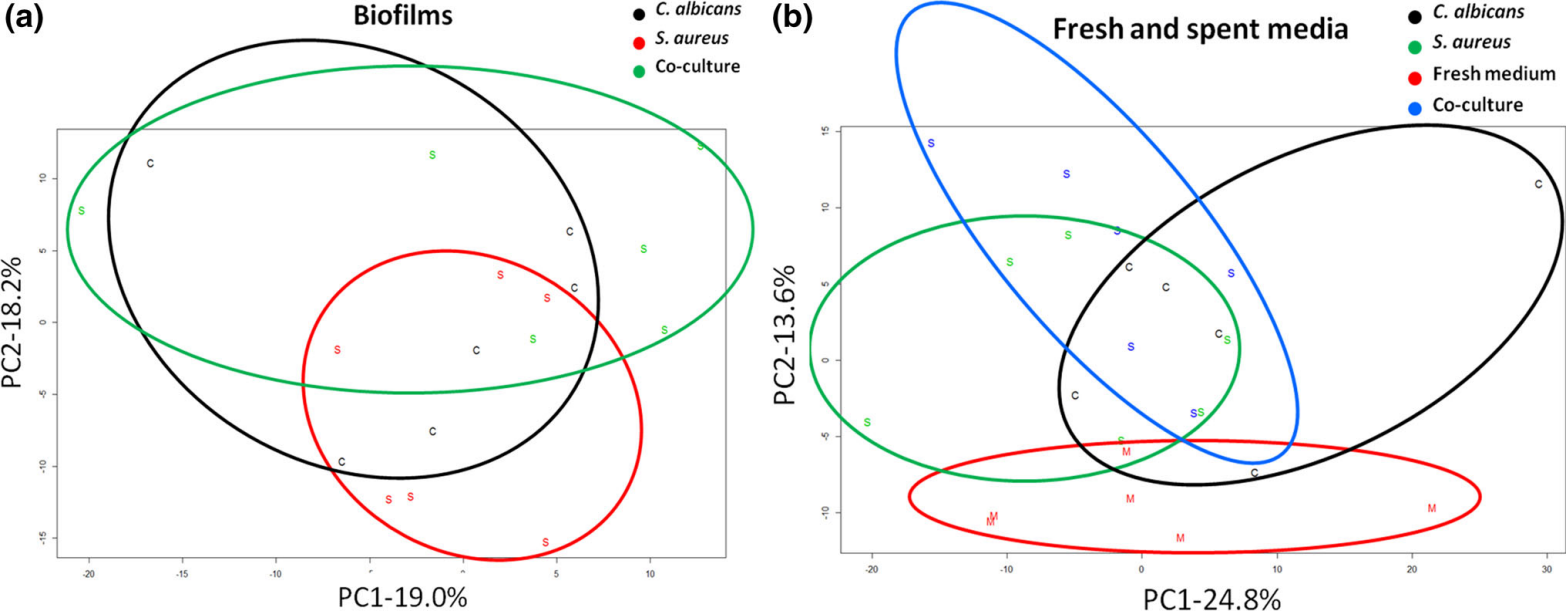
PCA of biofilms (**a**) and spent and fresh medium (**b**). In **a** the intracelullar metabolome from *Candida albicans* mono-culture is shown in *black*, the intracellular metabolome from *Staphylococcus aureus* mono-culture is shown in *red*, and the intracelullar metabolome of the co-culture is shown in *green*. In **b** the extracellular metabolome from *Candida albicans* mono-culture is shown in *black*, the extracellular metabolome from *Staphylococcus aureus* mono-culture is shown in *green*, fresh medium is shown in *red* and the extracellular metabolome from the co-culture is shown in *blu*e. Note that in the biofilms, there is significant overlap between all datasets, likely due to a combination of noise and the similarity of intracellular components of both organism. In the medium, however, **b** there is considerable differentiation between the fresh medium and the bacterial and fungal cultures, while there is considerable overlap of the spent media. The co-culture, as is to be expected, forms the middle point between the clusters of spent medium. Overall, the contribution of the first two principle components in both sample sets is modest (at 19 and 24.8 % for PC1 in the biofilms and media respectively). This is consistent with the presence of considerable background chemical noise in the data

Analysis of the cell extracts resulted in 41 identifications detected using the targeted method (see Supplementary Data tables S1 and S2). PCA of the biofilm samples using the XCMS/MzMatch/IDEOM software (Fig. 5a) showed significant overlap between co- and mono-cultures in PCs 1 and 2. PCA of the media (Fig. 5b) showed clearer separation of the fresh medium from all spent media. Univariate statistical analysis was therefore applied to the dataset to elucidate key differences between the samples.

Cholesterol is consumed in *S. aureus*, but is excreted into the medium in *C. albicans* and co-culture biofilms. A recent study showed that a pathogenic strain of *S. aureus*, responsible for bovine mastitis in dairy cows, consumed cholesterol and sterol esters when incubated in milk (Vidanarachchi et al. 2015). Various bacteria can utilize cholesterol as a carbon source for growth (Arima et al. 1969; Nagasawa et al. 1969) and it has since been suggested that steroid intermediates of the bacterial cholesterol degradation pathway in some pathogenic bacterial species contribute to their pathogenicity and survival within the host (Klink et al. 2013; Yam et al. 2011; Petrusma et al. 2014).

An interesting result from the cells is the significant pooling of sedoheptulose-7-phosphate in the co-culture, at much higher levels than was observed in either of the monocultures. As an intermediate in the pentose phosphate pathway, one would expect other compounds in the same pathway to be up- or down-regulated, but this is not observed, with a pattern of low levels of sugar phosphate in the *S. aureus* samples, and equivalent levels in *C. albicans* and co-culture cells, except for ribose-5-phosphate. One explanation for the accumulation of S7P in the co-culture cells could be the effect that elevated sugar phosphate concentrations have on bacterial growth. In a previous study, *E. coli* grown in the presence of sugar phosphates that could be directly incorporated into the glycolytic pathway resulted in cell death, whereas those grown in the presence of sugar phosphates that were not directly incorporated into glycolysis merely resulted in growth inhibition (Kadner et al. 1992). It could be suggested that *C. albicans* is secreting S7P, possibly in conjunction with another metabolite that can overcome catabolite repression, (Ackerman et al. 1974), to control the growth of *S. aureus* in the co-cultured biofilm.

**Fig. 6:**
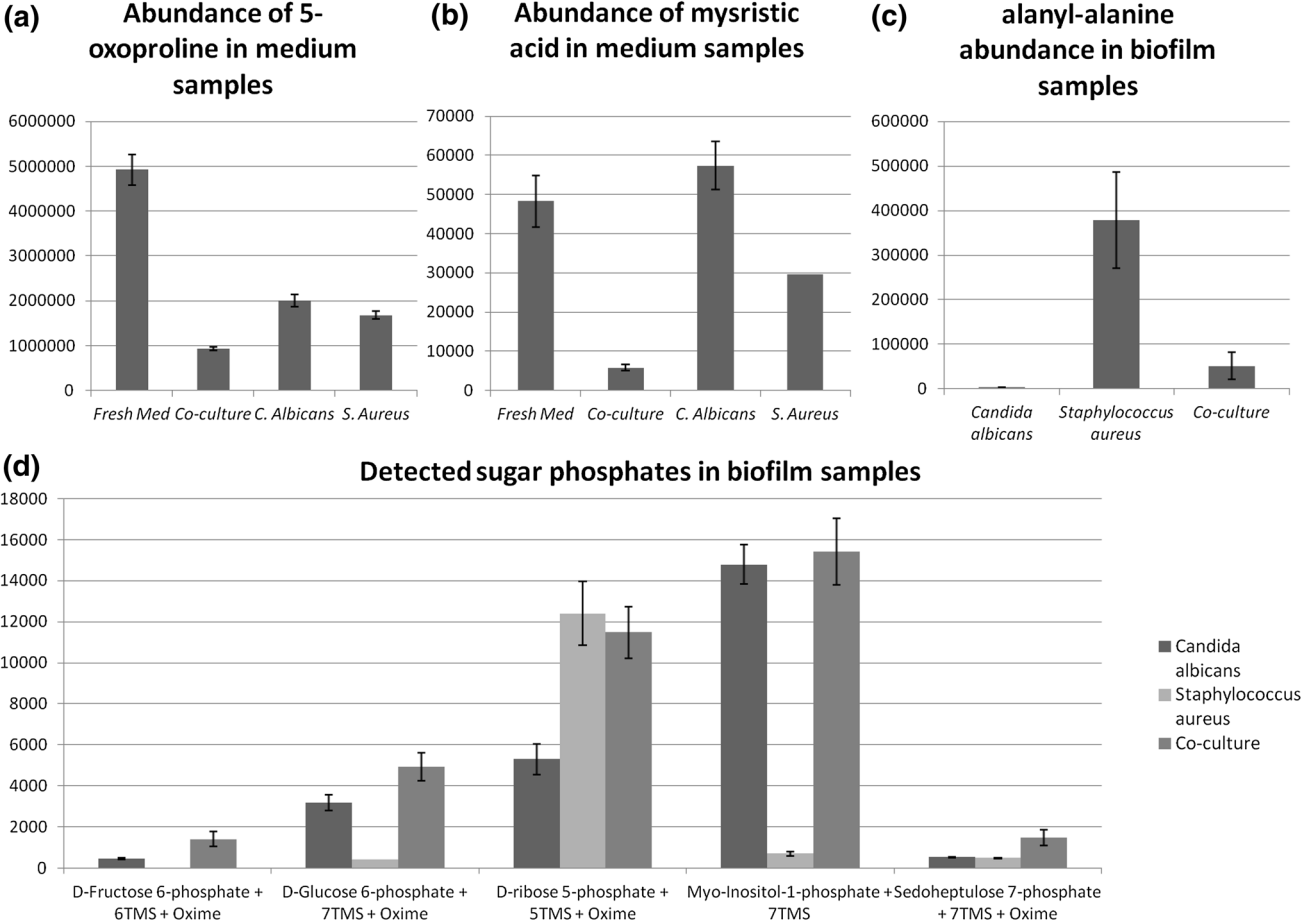
**a** Abundance of 5-oxoproline (validated from untargeted data) in the medium. The significant drop in abundance between fresh medium and all cultures demonstrates its uptake. This is particularly marked in the co-culture. **b** Myristic acid (validated from untargeted data) is not consumed by *C.albicans* but is significantly consumed in the co-culture. A single *S. aureus* culture showed significant levels (~30,000 counts) but was not detected in the 5 other replicates, precluding the addition of *error bars*. **c** Detection of alanyl alanine in the biofilm samples. It is markedly higher in the *S. aureus* cells, probably as a consequence of its function in cell wall biosynthesis. **d** Intensities of sugar phosphates detected within the cell/biofilm pellets. *C. albicans* monoculture values are shown in *dark grey*, *S. aureus* monoculture values are shown in *light grey*, and co-culture values are shown as *mid-grey*. Values are shown with standard error of the mean

Untargeted analysis, processed using our in-house pipeline, resulted in the detection of 465 compounds from medium and 405 from cells. A shortlist of the most significantly changing metabolites was selected and analysed for high quality fragment pattern matches. Three compounds were chosen for validation: two from medium and one from intracellular samples. Pyroglutamate (5-oxoproline) was consumed in all cultures in comparison to medium (see Fig. 6a). This is the same pattern seen for the proteinaceous amino acids and may well function, via the action of 5-oxoprolinase, as an additional source of glutamic acid (Kumar and Bachhawat 2011). Myristic acid, found to be consistently depleted from the medium in the *S. aureus* containing samples (see Fig. 6b), is known to be bacteriocidal, and depletion may be a consequence of detoxification (Liu and Huang 2012; Kitahara et al. 2004).

Alanyl-alanine is dramatically upregulated (132-fold) in the intracellular metabolome of *Staphylococcus aureus* as compared to *Candida albicans* (see Fig. 6c), although the expression drops to 17-fold in the co-culture. Our current methodology is unable to distinguish chiral enantiomers, and therefore it is highly likely that this upregulation is due to the synthesis of the D-Ala-D-Ala linker dipeptide, a component of the mucopeptide precursor in *Staphylococcus aureus* (Francis 1962).

Interestingly, targeted detection of sugar phosphates was prevalent in the cell samples (Fig. 6d) but only one, myo-inositol 1 phosphate, was detected in the medium samples. This is consistent with a lack of cell death and proliferation of the cells, as sugar phosphate leakage is associated with cell rupture (Rajendran et al. 2014).

## Conclusion

Gas chromatography mass spectrometry remains a powerful technique for metabolomics analysis. The ability to distinguish isomeric compounds in a broad-based analysis using retention time is very useful for the study of metabolism. Acquiring data using the Q Exactive GC operated in full scan at high resolving power allows for more compounds to be analyzed, increasing the scope of the analysis. The advantage of such technique is that data processing can be split into a targeted (using a compound database) and untargeted (unknown compound discovery) workflow and the possibility to historically re-interrogate the data at a later stage if needed. Data from mono- and co-cultured *C. albicans* and *S. aureus* strains demonstrates the utilisation of specific sugars as a carbon source in both organisms in this intricate interkingdom interaction, as well as synergistic effects on intermediates in the pentose phosphate pathway. This suggests that targeting the enzymes associated with sedoheptulose-7-phosphate may have an effect on the metabolic interaction between *C. albicans* and *S. aureus*. The ability to harness sophisticated technologies as those described herein has immense potential in the study of complex microbiome studies where the interaction of the microbiome with the mycobiome have implication for human health and disease.

## Electronic supplementary material

Below is the link to the electronic supplementary material.

Supplementary material 1 (DOCX 235kb)

Supplementary material 2 (XLSX 11383 kb)

Supplementary material 3 (XLSX 11516 kb)
