# Supplementary material for: A novel targeted/untargeted GC-Orbitrap metabolomics methodology applied to Candida albicans and Staphylococcus aureus biofilms
Source: Metabolomics. 2016 Nov 5;12(12):189. doi: 10.1007/s11306-016-1134-2 (PMC5097782; doi:10.1007/s11306-016-1134-2)
Supplement: Supplementary file 1 — Supplementary material 1 (DOCX 235kb) [file 11306_2016_1134_MOESM1_ESM.docx]

# Supplementary Methods

## Sample preparation

Retention index mix was prepared from pure alkanes dissolved in hexane to a final concentration of 6 mg/ml. Stock solutions were prepared for each from neat reference standard in water or HCl acidified water for amino acids insoluble in water alone. A custom standard mixture of sugars, sugar phosphates, pentose phosphates and amino acids were then prepared by mixing the stock solutions together and diluting with water. A volume of 30 µL extracted sample, as well as each standards mix of sugars, sugar phosphates, pentose phosphate pathway, and amino acids, were transferred into a 300 µL KIMSHIELD™ deactivated glass polyspring insert (National Scientific). Internal standards ^13^C_6_-Glucose (2 nmol), D_27_-Myristic Acid (2 nmol) and Scyllo-Inositol (1 nmol) were added to each sample. Samples were then dried in a Savant SPD1010 SpeedVac concentrator (Thermo Scientific) for 90 min. Inserts were then placed into a 9mm screw cap amber borosilicate glass 1.5 mL vial (Thermo Scientific). 50 µL of 20 mg / ml (w/v) methoxyamine HCl in pyridine was added to each dried sample and sealed. The vial and insert were vortexed for 10 seconds and incubated at 60 °C for 120 min. Following the methoximation step, 50 µL of MSTFA + 1 % TMCS ( N-Methyl-N-(trimethylsilyl) trifluoroacetamide + 1 % trimethylchlorosilane) was added, followed by a further 10 second of vortexing. Silylation was performed by incubation at 80°C for a further 120 min. Samples were cooled to room temperature. 1 µL of retention index alkane mixture was added to each sample. Samples were then ready for injection.

## Instrument parameters

The acquisition sequence started with six injections of a matrix conditioning samples prior to running the biofilm and standards mix samples. Derivatized samples, reference standards mixes and QC samples were injected in randomised order across the batch, for a total of 57 injection runs. A robotic arm (Thermo Scientific™ TriPlus™ RSH autosampler ) injected 1µL of derivatized sample into a split/splitless (SSL) injector at 250 °C using a 1:100 split flow on a Thermo Scientific™ TRACE™ 1310 GC. Helium carrier gas at a flow rate of 1.0 mL/min was used for separation on a TraceGOLD TG-5SILMS 30 m length × 0.25 mm inner diameter × 0.25 µm film thickness column (Thermo Scientific). The initial oven temperature was held at 70 °C for four minutes, followed by an initial gradient of 20 °C/min ramp rate. The final temperature was 320 °C and held for eight minutes. Eluting peaks were transferred through an auxiliary transfer temperature of 250 °C into the QExactive-GC mass spectrometer (Thermo Scientific). Electron ionisation (EI) at 70 eV energy, emission current of 50 µA with an ion source temperature of 230 °C was used in all experiments. A filament delay of 5.3 min was selected to prevent excess reagents from being ionised. High resolution EI fragment spectra were acquired using 60,000 resolution (FWHM at *m/z* 200) with a mass range of 50-650 *m/z.* The best internal lock mass from *m/z* 207.0324, 281.0511 or 355.0699 was used to maintain mass accuracy within 1 ppm throughout the chromatogram.

## Comparison with ITQ-900

Inserts were then placed into a 9mm screw cap amber borosilicate glass 1.5 mL vial. 50 µL of 20 mg / ml (w/v) methoxyamine HCl in pyridine was added to each dried sample and sealed. The vial and insert were vortexed for 10 seconds and incubated at 80 °C for 15 min. Following the methoximation step, 50 µL of MSTFA + 1 % TMCS ( N-Methyl-N-(trimethylsilyl) trifluoroacetamide + 1 % trimethylchlorosilane) was added, followed by a further 10 second of vortexing.  Silylation was performed by incubation at 80°C for a further 15 min. 1 µL of TMS derivatized extract was injected on both an ITQ 900 (Thermo Scientific) coupled to a Trace Ultra GC with PTV vaporiser and the QExactive-GC, with an SSL vaporiser. Both GC system parameters were as follows; A 1:10 split ratio using a helium carrier flow of 1 mL/min, with vaporiser temperature of 280 °C.  Initial oven temperature was 70 °C and was held for 4 min, with a final oven temperature of 300 °C using a temperature gradient ramp rate of 20 °C/min.  The maximum oven temperature was held for a further 4 min to condition the column before returning to initial conditions.  The MS transfer line temperature was 240 °C. The EI source temperature was 250 °C using 70 eV electron energy for ionisation.

## Data processing

Acquired data were processed using a targeted compound list based on the measured EI fragment from authentic standards. The exact mass of the most abundant fragment along with the measured retention time for each compound was entered into a compound database in TraceFinder 4.0 (Thermo Scientific, Runcorn, UK). Compound databases were verified using the standards mix chromatograms acquired during the batch. The data from the samples were then screened against the compound databases and the integrated area for each detected compound recorded. Peak areas were then normalised using total detected signal to compensate for differences in sample loading. The normalised areas were then log2 transformed and compared. Compounds detected using this methodology were matched to data obtained on the same instrument using authentic standards and can therefore be considered MSI category 1 (identifications) and are listed in the supplementary tables (S1 and S2) as ‘targeted’.

Untargeted screening was performed using the XCMS/MzMatch/IDEOM pipeline (Creek et al. 2012) for unknown compound discovery. Parameters used were standard orbitrap parameters as described in (Creek et al. 2012). The ‘related peaks’ step efficiently clusters compounds with similar peak shape (Pearson correlation of 0.7 or above) and retention time, setting the highest intensity as a ‘base peak’. The base peak lists were grouped, relatively quantified and PCAs were produced using IDEOM’s comparison module. Significantly modulated compounds were selected, searched against NIST (2014) and the Coon lab HRMS library, and three compounds were selected for validation on this basis. Standards were purchased from Sigma-Aldrich (Dorset, UK) and injected along with a sample predicted to have the compound in high concentration. Matches were confirmed using both fragment pattern and retention time (Figs S1-S3).

# Supplementary Data

Table S1: Table of log2 fold change of detected metabolites, classified by compound type. SAC samples are *Staphylococcus aureus* biofilm cultures. CAC are *Candida albicans* biofilms, SCC are *Staphylococcus/Candida* co-culture biofilms. All comparisons are against *Candida albicans* biofilms as a baseline, e.g. L-Cysteine is downregulated 10^2^ fold in *S. aureus* comparison to *Candida*.

| **Compound ID (NIST)** | **SAC vs CAC** | **SCC vs CAC** | **Detection method** | **Compound type** |
| --- | --- | --- | --- | --- |
| L-Cysteine, 3TMS | -10.2 | -0.6 | Targeted | Amino acid |
| L-Histidine, 3TMS | -6.4 | -1.0 | Targeted | Amino acid |
| L-Methionine, 2TMS | -3.2 | -1.2 | Targeted | Amino acid |
| L-Tyrosine, 3TMS | -2.8 | -0.8 | Targeted | Amino acid |
| L-Homoserine, 3TMS | -2.5 | 0.9 | Targeted | Amino acid |
| L-Threonine, 3TMS | -2.2 | -0.6 | Targeted | Amino acid |
| L-Glutamic acid, 3TMS | 1.9 | 0.3 | Targeted | Amino acid |
| L-Tryptophan, 3TMS | -1.9 | -0.5 | Targeted | Amino acid |
| L-Lysine, 3TMS | -1.5 | -0.4 | Targeted | Amino acid |
| L-Aspartic Acid, 3TMS | 1.4 | -0.4 | Targeted | Amino acid |
| L-Leucine, 2TMS | -1.4 | -0.5 | Targeted | Amino acid |
| L-Serine, 3TMS | -1.2 | -0.1 | Targeted | Amino acid |
| L-Proline, 2TMS | -0.9 | -1.1 | Targeted | Amino acid |
| L-Isoleucine, 2TMS | -0.9 | 0.3 | Targeted | Amino acid |
| L-Ornithine (and L-Argininine), 3TMS | 0.8 | 0.1 | Targeted | Amino acid |
| L-Alanine, 2TMS | -0.8 | -1.0 | Targeted | Amino acid |
| L-Valine, 2TMS | -0.7 | -0.1 | Targeted | Amino acid |
| L-Phenylalanine, 2TMS | 0.4 | 0.5 | Targeted | Amino acid |
| L-Hydroxyproline, 3TMS | -0.3 | -0.2 | Targeted | Amino acid |
| Glycine_3TMS | 0.1 | 0.1 | Targeted | Amino acid |
| Palmitic Acid, TMS | 0.1 | 0.2 | Targeted | Fatty acid |
| Octadecanoic Acid (Stearic Acid), TMS | 0.1 | 0.1 | Targeted | Fatty acid |
| Lactic Acid, 2TMS | -1.5 | -0.4 | Targeted | Organic acid/energy metabolism |
| Succinic acid, 2TMS | -1.1 | -0.9 | Targeted | Organic acid/energy metabolism |
| Cholesterol, TMS | 1.0 | 0.7 | Targeted | Steroid |
| D-Fructose, 5TMS + Oxime | 0.9 | -0.3 | Targeted | Sugar |
| D-Glucose, 6TMS + Oxime | -0.5 | -0.9 | Targeted | Sugar |
| D-Ribose + 4TMS + Oxime | -15.5 | -0.5 | Targeted | Sugar |
| L-Rhamnose + 4TMS + Oxime | 12.9 | 10.8 | Targeted | Sugar |
| D-Erythrose + 4TMS + Oxime | -9.2 | 1.1 | Targeted | Sugar |
| Maltose + 8TMS + Oxime | -4.3 | -0.7 | Targeted | Sugar |
| D-Xylulose + 4TMS + Oxime | -2.5 | 0.6 | Targeted | Sugar |
| Myo-Inositol + 6TMS | -1.7 | -0.3 | Targeted | Sugar |
| Sucrose + 8TMS | 0.7 | 0.6 | Targeted | Sugar |
| D-Mannose + 5TMS + Oxime | -0.7 | -0.9 | Targeted | Sugar |
| D-Lactose + 8TMS + Oxime | 0.2 | -1.1 | Targeted | Sugar |
| Adonitol + 5TMS | -4.3 | -0.5 | Targeted | Sugar alcohol |
| D-Sorbitol + 6TMS | -1.4 | -1.0 | Targeted | Sugar alcohol |
| D-Glucose 6-phosphate + 7TMS + Oxime | -11.5 | 0.8 | Targeted | Sugar phosphate |
| D-Fructose 6-phosphate + 6TMS + Oxime | -8.7 | 1.6 | Targeted | Sugar phosphate |
| Myo-Inositol-1-phosphate + 7TMS | -4.4 | 0.2 | Targeted | Sugar phosphate |
| D-ribose 5-phosphate + 5TMS + Oxime | 1.4 | 1.3 | Targeted | Sugar phosphate |
| Sedoheptulose 7-phosphate + 7TMS + Oxime | 0.0 | 10.3 | Targeted | Sugar phosphate |

Table S2: Table of log2 fold change of detected metabolites, classified by compound type. SAM samples are *Staphylococcus aureus* spent media. CAM are *Candida albicans* spent media, SCM are *Staphylococcus/Candida* co-culture spent media. All comparisons are against fresh medium (MO) as a baseline, e.g. L-Lysine is downregulated 5.7^2^ fold in *S. aureus* comparison to fresh medium.

| **compound ID (NIST)** | **CAM vs MO** | **SAM vs MO** | **SCM vs MO** | **Detection method** | **Compound Type** |
| --- | --- | --- | --- | --- | --- |
| Glycine_3TMS | 0.0 | 0.1 | 0.0 | Targeted | Amino acid |
| L-Alanine, 2TMS | -1.0 | -0.4 | -0.7 | Targeted | Amino acid |
| L-Aspartic Acid, 3TMS | -1.0 | -0.8 | -2.6 | Targeted | Amino acid |
| L-Cysteine, 3TMS | 0.3 | -1.6 | 0.1 | Targeted | Amino acid |
| L-Glutamic acid, 3TMS | -1.9 | -0.9 | -3.6 | Targeted | Amino acid |
| L-Histidine, 3TMS | -2.1 | -0.2 | -1.1 | Targeted | Amino acid |
| L-Homoserine, 3TMS | -0.2 | 3.0 | 2.7 | Targeted | Amino acid |
| L-Hydroxyproline, 3TMS | 0.1 | 0.2 | 0.3 | Targeted | Amino acid |
| L-Isoleucine, 2TMS | -1.5 | -3.6 | -2.9 | Targeted | Amino acid |
| L-Leucine, 2TMS | -1.7 | -2.8 | -3.3 | Targeted | Amino acid |
| L-Lysine, 3TMS | ND | 0.3 | ND | Targeted | Amino acid |
| L-Methionine, 2TMS | -6.8 | -6.0 | ND | Targeted | Amino acid |
| L-Ornithine (and L-Argininine), 3TMS | -0.8 | -0.7 | -0.8 | Targeted | Amino acid |
| L-Phenylalanine, 2TMS | -1.0 | -2.1 | -2.2 | Targeted | Amino acid |
| L-Proline, 2TMS | -6.3 | -1.0 | -3.9 | Targeted | Amino acid |
| L-Serine, 3TMS | -1.8 | -2.8 | -3.3 | Targeted | Amino acid |
| L-Threonine, 3TMS | -2.0 | -3.5 | -4.5 | Targeted | Amino acid |
| L-Tryptophan, 3TMS | -0.3 | -2.0 | -1.3 | Targeted | Amino acid |
| L-Tyrosine, 3TMS | 0.0 | -5.1 | -1.7 | Targeted | Amino acid |
| L-Valine, 2TMS | -0.7 | -2.0 | -1.8 | Targeted | Amino acid |
| Octadecanoic Acid (Stearic Acid), TMS | 0.0 | -0.1 | -0.1 | Targeted | Fatty acid |
| Palmitic Acid, TMS | 0.0 | -0.1 | -0.1 | Targeted | Fatty acid |
| Lactic Acid, 2TMS | 0.1 | -0.1 | -0.8 | Targeted | Organic acid/energy metabolism |
| Succinic acid, 2TMS | 0.2 | 0.4 | 0.3 | Targeted | Organic acid/energy metabolism |
| Cholesterol, TMS | 0.1 | 0.0 | -0.2 | Targeted | Steroid |
| D-Glucose, 6TMS + Oxime | -6.2 | -8.1 | -7.5 | Targeted | Sugar |
| D-Fructose, 5TMS + Oxime | -7.2 | -8.1 | -7.8 | Targeted | Sugar |
| 2-Deoxy-D-glucose + 4TMS + Oxime | 0.2 | 0.0 | 0.1 | Targeted | Sugar |
| D-Erythrose + 4TMS + Oxime | -0.6 | 0.2 | -1.1 | Targeted | Sugar |
| D-Ribose + 4TMS + Oxime | 0.1 | -0.2 | -0.2 | Targeted | Sugar |
| D-Xylulose + 4TMS + Oxime | -0.4 | 0.5 | 0.3 | Targeted | Sugar |
| Fucose + 4TMS + Oxime | 0.0 | 0.0 | -0.1 | Targeted | Sugar |
| Maltose + 8TMS + Oxime | 0.6 | -1.6 | -1.7 | Targeted | Sugar |
| Myo-Inositol + 6TMS | 0.0 | 0.0 | -0.1 | Targeted | Sugar |
| Sucrose + 8TMS | 2.1 | -0.2 | 0.0 | Targeted | Sugar |
| Adonitol + 5TMS | 1.1 | 0.2 | 1.0 | Targeted | Sugar alcohol |
| D-Sorbitol + 6TMS | 0.0 | -0.2 | -0.2 | Targeted | Sugar alcohol |
| Myo-Inositol-1-phosphate + 7TMS | 0.4 | 0.4 | 0.6 | Targeted | Sugar phosphate |

Table S3: List of derivatized internal standard compounds added to each sample prior to analysis. The elemental formula is calculated for most abundant EI fragment. For each compound the monoisotopic exact mass of the M+· ion calculated from the formula, along with the measured retention time and retention time window used for identification is shown.

| Compound Name | base peak Elemental Formula | Monoisotopic Mass M+· | RT (min) | Window  (s) |
| --- | --- | --- | --- | --- |
| 13C6-Glucose, 6TMS + oxime | [13]C4C9H31O3Si3 | 323.1710 | 12.66 | 5 |
| 27D-Myristic Acid + TMS -CH3 + H2O | C16D27H8O3Si | 330.4045 | 12.32 | 5 |
| scyllo-Inositol, 6TMS | C13H30O3Si3 | 318.1497 | 13.34 | 5 |

Table S4: List of sugars, sugar phosphates and pentose phosphate pathway derivatized standards used for targeted analysis. The elemental formula is calculated for most abundant EI fragment. For each compound the monoisotopic exact mass of the M+· ion calculated from the formula, along with the measured retention time and retention time window used for identification is shown.

| Compound Name | base peak Elemental Formula | Monoisotopic Mass M+· | RT (min) | Window  (s) |
| --- | --- | --- | --- | --- |
| 2-Deoxy-D-glucose + 4TMS + Oxime | C9H21O2Si2 | 217.1075 | 12.08 | 5 |
| 2-deoxy-ribose + 3TMS + Oxime | C9H21O2Si2 | 217.1075 | 10.82 | 5 |
| 2-phosphoglycerate + 4TMS | C14H36O7PSi4 | 459.1270 | 11.99 | 5 |
| 3-phosphoglycerate + 4TMS | C14H36O7PSi4 | 459.1270 | 12.15 | 5 |
| 6-Phosphogluconic acid + 7TMS | PO4Si3C9H28 | 315.1028 | 14.68 | 5 |
| Adonitol + 5TMS | C9H21O2Si2 | 217.1075 | 11.72 | 5 |
| D_Threose + 4TMS + Oxime | C8H21O2Si2 | 205.1075 | 10.08 | 3 |
| D-Arabinose + 4TMS + Oxime | C9H21O2Si2 | 217.1075 | 11.34 | 3 |
| D-Erythrose + 4TMS + Oxime | C8H21O2Si2 | 205.1075 | 10.04 | 3 |
| D-Erythrose 4-phosphate +5TMS | C11H30O5PSi3 | 357.1133 | 12.69 | 5 |
| D-Fructose 1,6-bisphosphate + 6TMS + Oxime | PO4Si3C9H28 | 315.1028 | 16.2 | 5 |
| D-Fructose 1-phosphate + 7TMS + Oxime | C12H36O4PSi4 | 387.1423 | 14.53 | 5 |
| D-Fructose 6-phosphate + 6TMS + Oxime | PO4Si3C9H28 | 315.1028 | 14.56 | 5 |
| D-Fructose, 5TMS + Oxime | C9H21O2Si2 | 217.1075 | 12.51 | 3 |
| D-Galactose + 5TMS + Oxime | C13H31O3Si3 | 319.1576 | 12.62 | 3 |
| D-Glucose 6-phosphate + 7TMS + Oxime | C12H36O4PSi4 | 387.1423 | 14.63 | 5 |
| D-Glucose, 6TMS + Oxime | C13H31O3Si3 | 319.1576 | 12.66 | 3 |
| Dihydroxyacetone phosphate + 3TMS + Oxime | C12H31NO6PSi3 | 400.1191 | 11.8 | 5 |
| D-Lactose + 8TMS + Oxime | C15H33O4Si3 | 361.1681 | 16.13 | 3 |
| D-Mannitol + 6TMS | C13H31O3Si3 | 319.1576 | 12.84 | 3 |
| D-Mannose + 5TMS + Oxime | C13H31O3Si3 | 319.1576 | 12.59 | 3 |
| D-Ribose + 4TMS + Oxime | C9H21O2Si2 | 217.1075 | 11.43 | 3 |
| D-ribose 5-phosphate + 5TMS + Oxime | PO4Si3C9H28 | 315.1028 | 13.66 | 5 |
| D-Sorbitol + 6TMS | C13H31O3Si3 | 319.1576 | 12.87 | 3 |
| Dulcitol + 6TMS | C9H21O2Si2 | 217.1075 | 12.9 | 3 |
| D-Xylose + 4TMS + Oxime | C9H21O2Si2 | 217.1075 | 11.3 | 3 |
| D-Xylulose + 4TMS + Oxime | C7H15NO2Si | 173.0867 | 11.43 | 5 |
| Fucose + 4TMS + Oxime | C5H13OSi | 117.0730 | 11.74 | 5 |
| L-Rhamnose + 4TMS + Oxime | C5H13OSi | 117.0730 | 11.68 | 5 |
| Maltose + 8TMS + Oxime | C15H33O4Si3 | 361.1681 | 16.32 | 3 |
| Myo-Inositol + 6TMS | C12H29O3Si3 | 305.1419 | 13.65 | 5 |
| Myo-Inositol-1-phosphate + 7TMS | C13H30O3Si3 | 318.1497 | 15.07 | 5 |
| Ribulose-5-phosphate + 5TMS + Oxime | C11H30O5PSi3 | 357.1133 | 13.7 | 5 |
| Sedoheptulose 7-phosphate + 7TMS + Oxime | C12H36O4PSi4 | 387.1423 | 15.59 | 5 |
| Sucrose + 8TMS | C15H33O4Si3 | 361.1681 | 15.92 | 3 |
| Xylitol + 4TMS + Oxime | C9H21O2Si2 | 217.1075 | 11.61 | 3 |

Table S5: Amino acid derivatized standards used for targeted analysis. The elemental formula is calculated for most abundant EI fragment. For each compound the monoisotopic exact mass of the M+· ion calculated from the formula, along with the measured retention time and retention time window used for identification is shown.

| Compound Name | base peak Elemental Formula | Monoisotopic Mass M+· | RT (min) | Window  (s) |
| --- | --- | --- | --- | --- |
| Glycine, 3TMS | C7H20NSi2 | 174.1129 | 8.44 | 5 |
| L-Alanine, 2TMS | C5H14NSi | 116.0890 | 7.17 | 5 |
| L-Asparagine, 4TMS | C9H23N2OSi2 | 231.1343 | 11.42 | 5 |
| L-Aspartic Acid, 3TMS | C9H22NO2Si2 | 232.1184 | 10.51 | 5 |
| L-Cysteine, 3TMS | C8H22NSSi2 | 220.1006 | 10.75 | 5 |
| L-Cystine, 4TMS | C8H20NO2Si2 | 218.1027 | 14.56 | 5 |
| L-Glutamic acid, 3TMS | C10H24NO2Si2 | 246.1340 | 11.12 | 5 |
| L-Glutamine, 3TMS | C7H14NOSi | 156.0839 | 12.01 | 5 |
| L-Histidine, 3TMS | C7H14N2Si | 154.0921 | 12.8 | 5 |
| L-Homoserine, 3TMS | C9H24NOSi2 | 218.1391 | 10.05 | 5 |
| L-Hydroxyproline, 3TMS | C10H24NOSi2 | 230.1391 | 10.56 | 5 |
| L-Isoleucine, 2TMS | C8H20NSi | 158.1360 | 8.93 | 5 |
| L-Leucine, 2TMS | C8H20NSi | 158.1360 | 8.76 | 5 |
| L-Lysine, 3TMS | C8H18NSi | 156.1203 | 11.65 | 5 |
| L-Methionine, 2TMS | C7H18NSSi | 176.0924 | 10.53 | 5 |
| L-Ornithine (and L-Arginine), 3TMS | C7H16NSi | 142.1047 | 11.11 | 5 |
| L-Phenylalanine, 2TMS | C11H18NSi | 192.1203 | 11.22 | 5 |
| L-Proline, 2TMS | C7H16NSi | 142.1047 | 8.99 | 5 |
| L-Serine, 3TMS | C8H22NOSi2 | 204.1234 | 9.42 | 5 |
| L-Threonine, 3TMS | C8H20NO2Si2 | 218.1027 | 9.61 | 5 |
| L-Tryptophan, 3TMS | C12H16NSi | 202.1047 | 14.25 | 5 |
| L-Tyrosine, 3TMS | C8H20NO2Si2 | 218.1027 | 12.91 | 5 |
| L-Valine, 2TMS | C7H18NSi | 144.1203 | 8.28 | 5 |

Table S6: Organic acid derivatized standards used for targeted analysis. The elemental formula is calculated for most abundant EI fragment. For each compound the monoisotopic exact mass of the M+· ion calculated from the formula, along with the measured retention time and retention time window used for identification is shown.

| Compound Name | base peak Elemental Formula | Monoisotopic Mass M+· | RT (min) | Window  (s) |
| --- | --- | --- | --- | --- |
| Lactic Acid, 2TMS | C8H19O3Si2 | 219.0867 | 6.65 | 5 |
| Methylmalonoic Acid, 2TMS | C9H19O4Si2 | 247.0816 | 8.25 | 5 |
| Octadecanoic Acid (Stearic Acid), TMS | C20H43O3Si | 359.2976 | 14.33 | 5 |
| Palmitic Acid, TMS | C18H39O3Si | 331.2663 | 13.42 | 5 |
| Succinic acid, 2TMS | C9H19O4Si2 | 247.0816 | 9.08 | 5 |
|  |  |  |  |  |


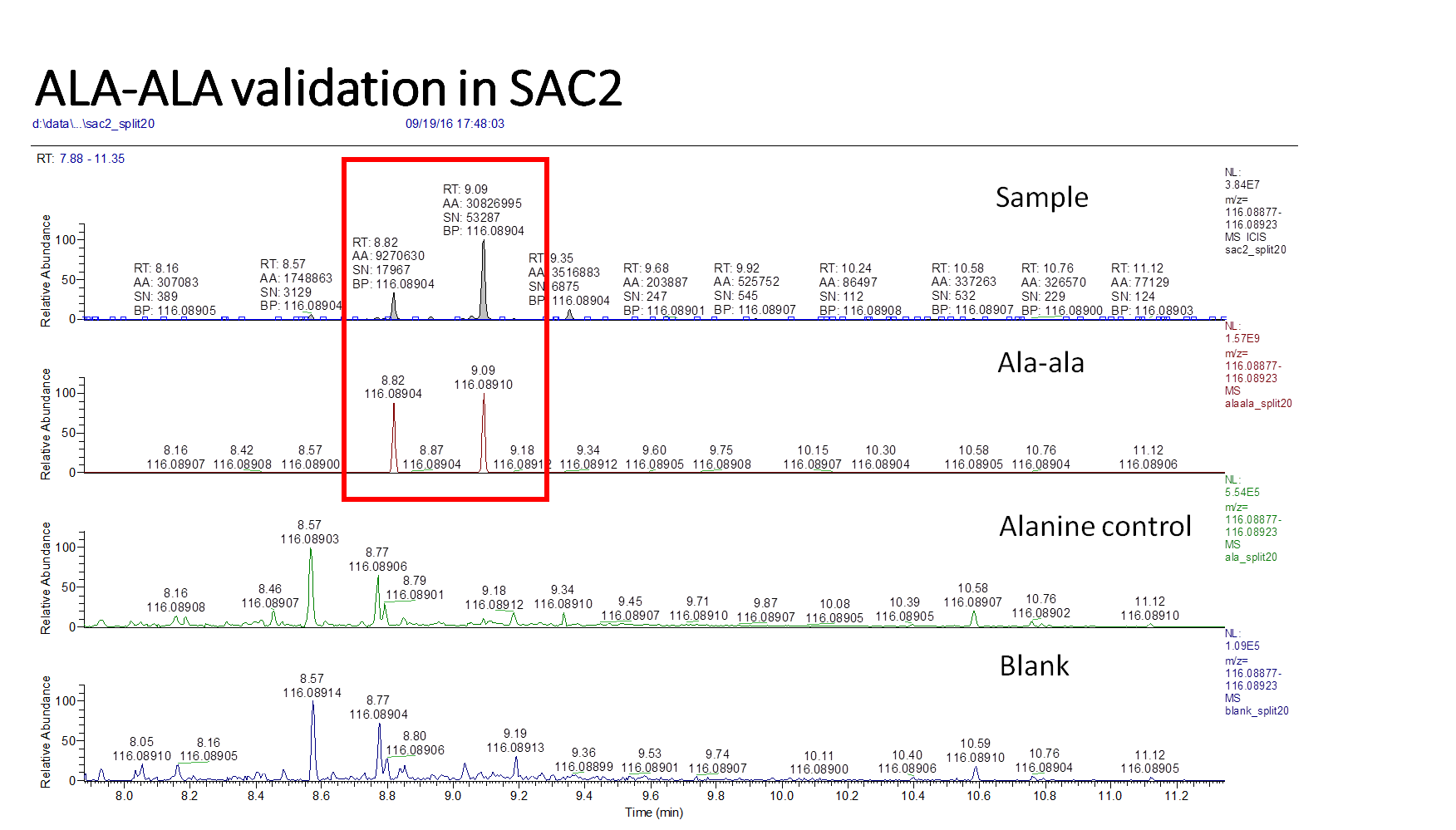


Figure S1: Validation data for the alanyl alanine dipeptide. Note the characteristic double peak in both the sample and standard. Note also that there is no overlap with the alanine control.


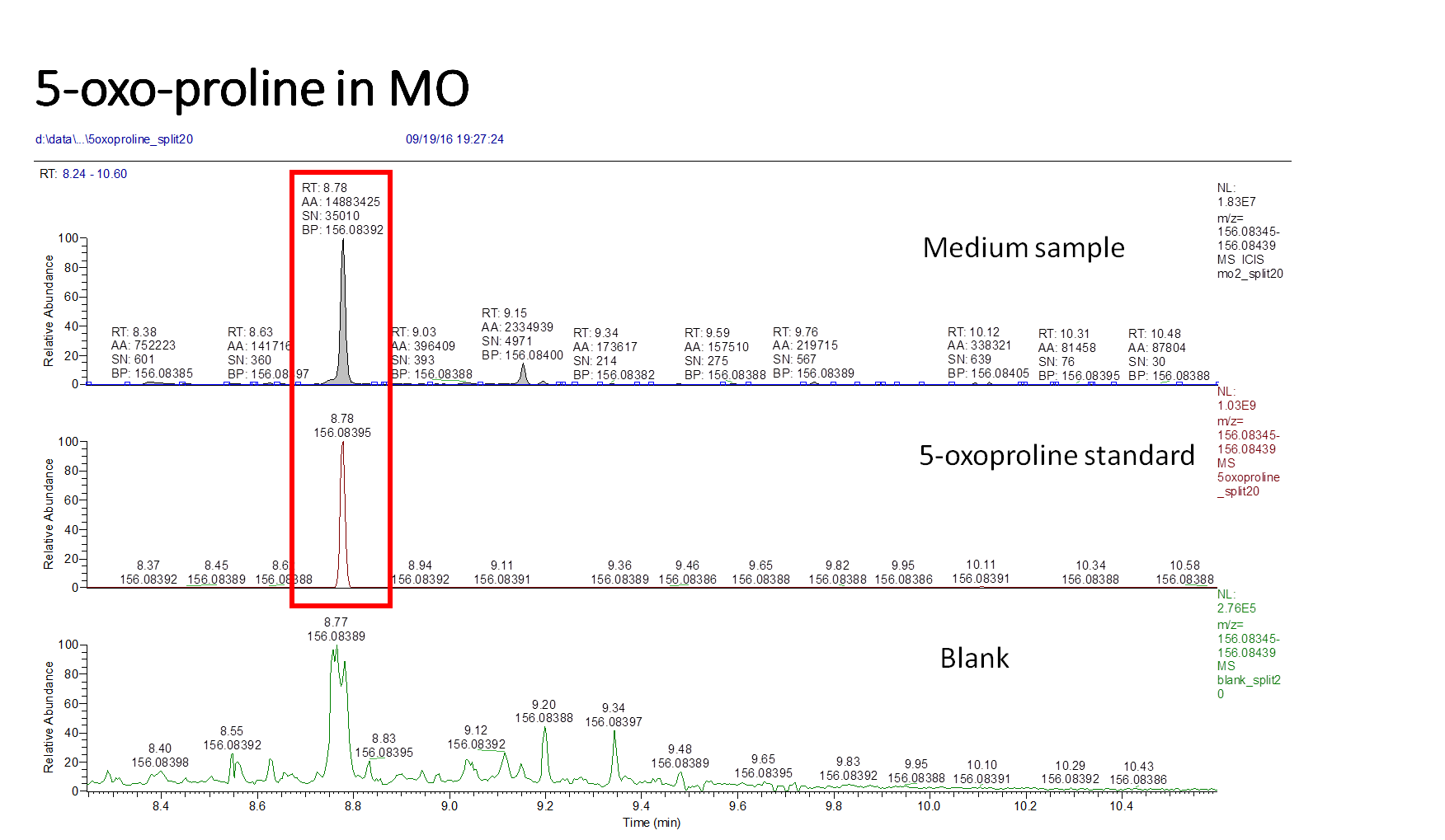


Figure S2: Validation data for 5-oxo-proline. Please note the strong peak in the medium and matching peak in the standard. A small peak is visible in the blank, probably due to carryover.


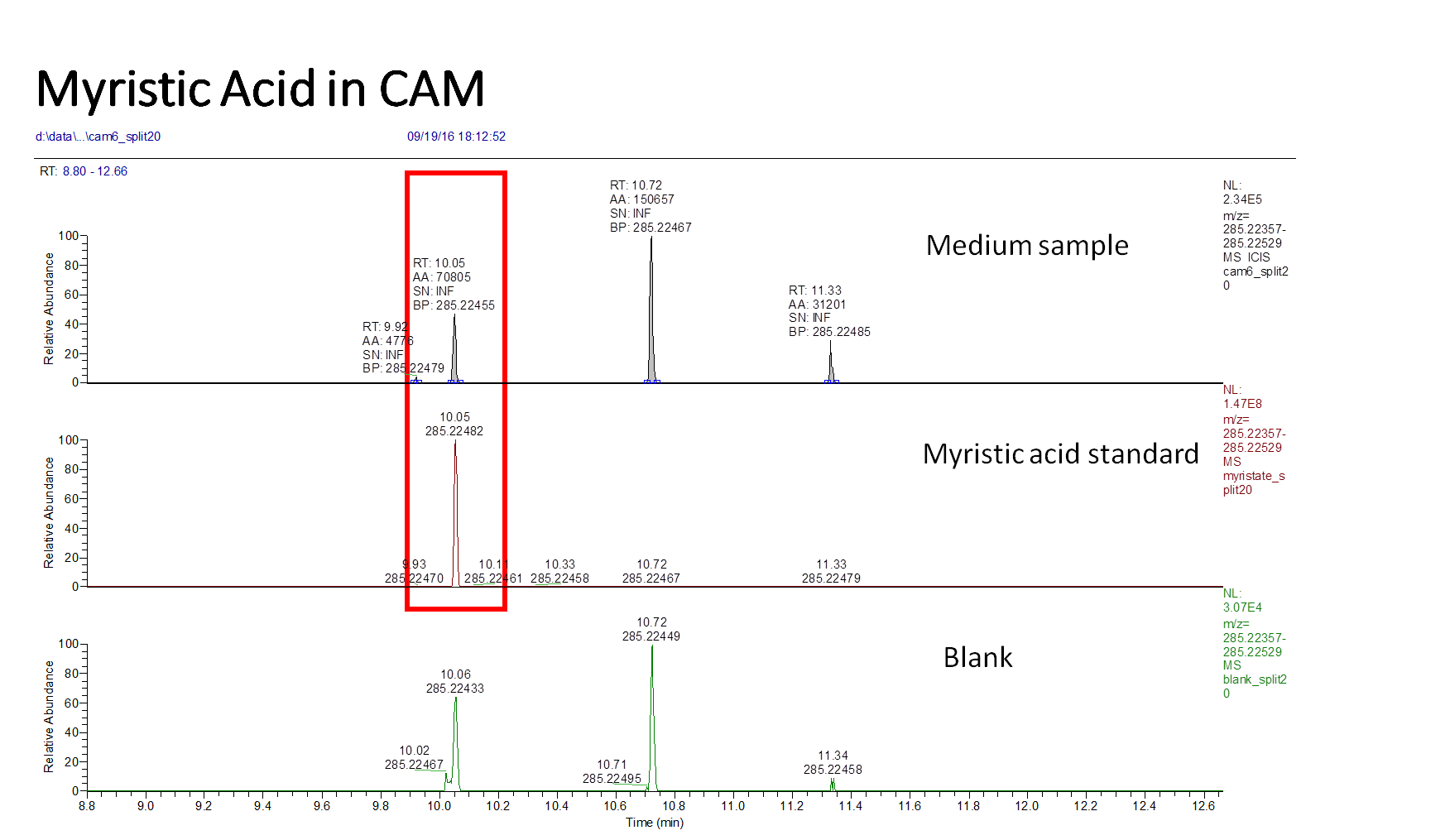


Figure 3: Validation data for myristic acid. Note the clear peak in the sample and standard. Again the very small peak in the blank is likely due to minor carryover.
